# Optimization of Xanthan and Locust Bean Gum in a Gluten-Free Infant Biscuit Based on Rice-Chickpea Flour Using Response Surface Methodology

**DOI:** 10.3390/foods10010012

**Published:** 2020-12-23

**Authors:** Soulef Benkadri, Ana Salvador, Teresa Sanz, Mohammed Nasreddine Zidoune

**Affiliations:** 1 Institut de la Nutrition, de l’Alimentation et des Technologies Agro-alimentaires (I.N.A.T.A-A.), Route Ain El bey, 25000 Constantine, Algeria; soulef.benkadri@umc.edu.dz (S.B.); zidounem@yahoo.fr (M.N.Z.); 2 Instituto de Agroquímica y Tecnología de Alimentos (IATA-CSIC), Avda. Agustín Escardino, 7, 46980 Paterna, Valencia, Spain; tesanz@iata.csic.es

**Keywords:** celiac children, gluten-free biscuit, xanthan gum, locust bean gum, RSM, rice chickpea flour

## Abstract

Incorporation of xanthan gum and locust bean gum in rice flour supplemented by chickpea flour was used to obtain a good quality of nutritionally enriched biscuit for celiac children. Response surface methodology (RSM) was applied to optimize the levels of xanthan and locust bean gum added to the composite gluten-free flour. Analysis was based on the rheological (hardness and viscoelastic) characteristics of the dough and specific volume, water activity, and hardness of the biscuit. The results revealed that the regression and variance analysis coefficients related to the rheological and physical properties of dough and biscuit under the influence of independent variables were sufficient for an adequate and well-fitted response surface model. Linear terms of variables significantly affect most of the dough and biscuit parameters, where the xanthan gum effect was found to be more pronounced than locust bean gum. Interaction terms showed a significant positive effect on the specific volume of the biscuits and a negative effect on the water activity. However, the interactive effect of gums did not significantly affect the rheological parameters of the dough. Optimized conditions were developed to maximize the specific volume of biscuit and minimize water activity and biscuit hardness, while keeping hardness and viscoelastic properties of the dough in range. Predicted responses were found satisfactory for both rheological and physical characteristics of dough and biscuit.

## 1. Introduction

Gluten-free cereals-based formulations are often low in nutrients, such as proteins, minerals, and other elements and display poor rheological properties of dough which make processing difficult and results in less desirable final product quality compared to wheat-based products.

The use of composite flours for gluten-free products is a recent development for economic and nutritional reasons, especially in developing countries, such as in North Africa, where wheat is the basic ingredient of most baked foods.

Field bean, dry pea, chickpea, and lentil in combination with rice or corn flours have been employed to substitute wheat flour in bread and pasta [1,2,3,4,5,6,7,8,9]. This substitution improves the nutritional properties of gluten-free products enhancing the health status of celiac patients with the additional advantage of using locally available legumes.

Hydrocolloids or gums have been widely used to improve the technological quality of leavened baked goods made from ingredients other than wheat flour. Whether natural (xanthan, carageenan, acacia, guar, tragacanth, and arabic gum) or synthetic (Hydroxypropylmethylcellulose-HPMC and carboxymethylcellulose-CMC), hydrocolloids’ ability to mimic the viscoelastic properties of gluten to improve dough handling and to improve the overall quality of finished baked products has been investigated [10,11,12,13,14,15,16,17,18].

In many cases, an individual hydrocolloid cannot provide the required functionality and a combination of two is required. When two hydrocolloids are mixed, their interactions profoundly affect food structural formation and consequently its texture, stability, and functionalities [16]. The use of such combinations was found in foods such as bakery and cereal products, where xanthan can act as a gelling agent in synergism with other gums. These gels are interesting because gelation of the mixtures occurs under conditions in which the individual components alone do not gel [19].

Xanthan gum (XG) is one of the most commonly used hydrocolloids in food products. XG is an anionic heteropolysaccharide of high molecular weight secreted by the bacterium *Xanthomonas campestris*. It consists of repeating units of D-glucose, linked to form the b-1,4-D-glucan cellulosic backbone. [20,21,22]. At low concentrations XG forms viscous solutions and its rheological behavior is very stable in a wide range of pH and temperature [23]. XG is a very effective thickener during dough preparation and stabilizer, helping in the retention of gas and the increase in the specific volume of bakery products [12,24,25,26,27,28,29,30,31].

The effect of binary gum mixtures in the quality of different food systems has been studied. Ahlborn et al. [32] reported that a formulation of rice bread containing XG and hydroxypropylmethylcellulose created a bi-continuous matrix with starch fragments, similar to gluten. Furthermore, XG is well known to yield a strong interaction with galactomannans, leading to useful thermo-reversible gels. Köksel [33] reported that XG–guar gum blend improved gluten-free cake quality with increasing specific volume as well as decreasing weight loss and crumb hardness values. In a study on gluten-free rice cakes, Sumnu et al. [34] reported that XG–guar gum blend was effective to retard cake staling.

Several authors have suggested models for gelation which differ in the details of the mode of intermolecular binding that is considered to occur. Certain studies [22,35] confirmed that intermolecular binding occurs between the denatured xanthan helix and the galactomannan.

The tendency for galactomannans to gel synergistically with XG seems to be sensitive to the mannose to galactose (MG) ratio of the galactomannan. Locust bean gum (LBG) (also known as carob gum) with MG 3.5 and tara gum (MG 2.7) form strong gels, whereas guar gum (MG 1.55) yields weaker gels [22].

LBG is a galactomannan extracted from the seed endosperm of the carob tree plant botanically known as *Ceratonia siliqua L.* It is very abundant in the Mediterranean region since ancient times and is currently produced in many Mediterranean countries such as Algeria. The most significant property of LBG is its ability to hydrate in hot water to give a viscous solution. It is generally less viscous than the galactomannan guar and tara gum. Its biodegradability, low toxicity, and low cost contribute to its increasing utilization in various fields [36].

The main objective of this research was to study the effect of XG and locust bean gum and their interaction on the rheological properties of gluten-free biscuit dough and in the final quality of biscuits made from rice–chickpea composite flour (R–CPF). Response surface methodology (RSM) was applied to determine the optimum levels of gum incorporation. The analysis was based on the rheological (Texture Profile Analysis-TPA and viscoelastic) characteristics of the dough and specific volume, water activity, and hardness of the biscuit.

## 2. Materials and Methods

### 2.1. Ingredients

The ingredients used to produce the biscuits were soft wheat flour (La Meta, S.A.U., Lleida, Spain), rice flour (La Meta, S.A.U., Lleida, Spain), chickpea flour (P B FOODS Ltd., Bradford, UK), sugar (DISEM, Torrente, Spain), salt, baking powder including: sodium bicarbonate (A. Martínez, Cheste, Spain) and ammonium hydrogen carbonate (VWR Prolabo Chemicals, Leuven, Belgium), water, vegetable-based shortening (Vandemoortele, Iberica ref 402666, Barcelona, Spain), xanthan gum (XG) (Satiaxane CX 911, Cargill, St-Germain-en-laye, France), and locust bean gum (LBG) (Bio-Industrie Maroc S.A, de Cargill Maroc, Casablanca, Maroc).

### 2.2. Experimental Plan

Response surface methodology was employed. It consists in designing experiments, selecting variables’ levels in experimental runs, fitting mathematical models, and finally selecting variables’ levels by optimizing the response. A central composite design (CCD) was used to design the experiments comprising of two independent variables (XG and LBG). A total of 13 combinations were generated (Table 1) and the experiments at the center point were repeated five times to calculate the repeatability of the method [37]. The parameters that influence dough and biscuit quality were taken as responses.

### 2.3. Biscuit Preparation

The basic biscuit formulation is given in Table 2. The dough and biscuit was prepared following the methodology previously used by Benkadri et al. [24].

### 2.4. Dough Measurements

#### 2.4.1. Hardness

A TA-XT.plus texture analyzer was used and the measurement conditions were the same used in Benkadri et al. [24]. Hardness is the maximum peak force from the force curve obtained (N).

#### 2.4.2. Linear Viscoelastic Properties

A controlled stress rheometer (AR-G2, TA-Instruments, Crawley, UK) was used following the methodology previously used by Benkadri et al. [24]. Frequency sweep tests from 0.01 to 10 Hz at a stress wave amplitude of 4 Pa (inside the linear region) were carried out. The storage modulus (G’), loss modulus (G”), and tan δ = G”/G’ were recorded.

### 2.5. Biscuit Evaluation

#### 2.5.1. Water Activity

The water activity (a_w_) was determined following the methodology previously used by Benkadri et al. [24].

#### 2.5.2. Specific Volume

The specific volumes were calculated as (thickness*width*length/weight) and expressed as cm^3^/g following the methodology previously used by Benkadri et al. [24].

#### 2.5.3. Hardness

Hardness of biscuits was measured following the methodology previously used by Benkadri et al. [24]. The Volodkevich bite upper jaw probe (VB) used simulates the action of an incisor tooth biting through food. The area under the curve (representing the hardness of the biscuit, in N.mm was calculated.

### 2.6. Data Analysis

The statistical software package (Minitab 8.1, 2017) was used to construct the experimental design and analyze the data. The experimental data obtained from the design were analyzed using the second order polynomial model given below:

Y = b_0_ + b_1_XG + b_2_LBG + b_11_ XG XG + b_22_ LBG LBG + b_12_ XG LBG (1)

where Y = response, XG, LBG = independent variables, and b_0_, b_1_, b_2_, b_11_, b_22_, b_12_ = regression coefficients.

Adequacy of the model was determined using coefficient of determination (𝑅^2^), F-value, and lack of fit. The effect of variables at linear, quadratic, and interactive levels on the response was described using various levels of significance. Response surface graphs were generated.

The optimization of the experimental parameters was done by the desirability function, which is a multicriteria numerical optimization technique which is very useful when it is necessary to find the best compromise between several responses.

Optimum values of the formulation variables were obtained after assigning certain constraints depending on the goals for each variable and response. Thus, Vsp was kept maximum while a_w_ was kept minimum. Dough hardness, the viscoelastic properties (G’ and G”), and biscuit hardness were kept in range.

## 3. Results and Discussion

### 3.1. Diagnostic Checking of the Models

Response surface analysis was performed to study the experimental data. The statistical significance of the model terms was examined with analysis of variance (ANOVA). The F-value was found significant for all models, implying that the models were accurate enough to predict the responses. Moreover, the F-values of the lack-of-fit test for all the models were insignificant, thus indicating that the experiments were carried out with adequate precision. R^2^ values for all the models were more than 0.94, which further validated the adequacy of models. All the models were statistically adequate and were used for studying the influence of processing variables on the various responses (Table 3).

### 3.2. Effect of Xanthan Gum (XG) and Locust Bean Gum (LBG) on Rice-Chickpea Composite Flour (R-CPF) Dough and Biscuit Properties

The results of regression analysis, depicted in Table 4, showed that the effects of processing variables on the dough and biscuit parameters were more significant at linear level than on quadratic level. Xanthan gum effect was found to be more pronounced than locust bean gum. Interaction between the gums showed a significant positive effect on the specific volume of the biscuits and a negative effect on the water activity. However, the interactive effect did not affect significantly the rheological parameters of the dough.

### 3.3. Dough Hardness

**Figure 1:**
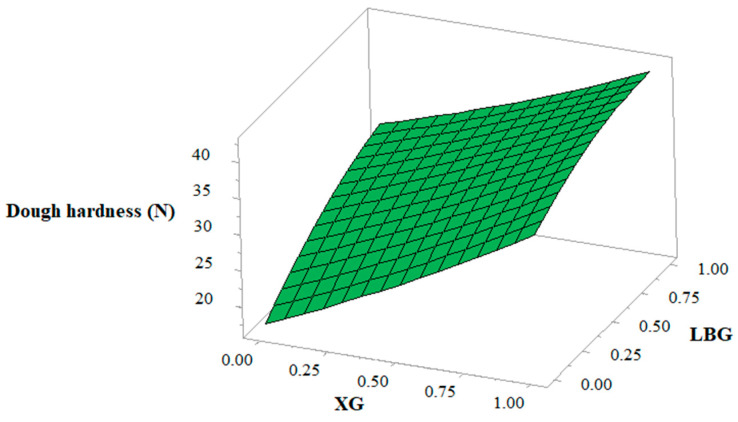
Response surface showing the effect of xanthan gum (XG) and locust bean gum (LBG) on dough hardness.

Hardness refers to the force required to compress the material up to a certain level. The coefficient of estimation of biscuit hardness showed that both gums had a significant (*p* < 0.001) linear effect on the hardness of dough, with the effect of XG being the more pronounced (Table 4). Several authors have studied the effect of different hydrocolloids on the rheological properties of dough in different gluten-free formulas. They reported that the greatest hardness was found in dough with added XG, as compared to the other hydrocolloids [25,27,30,38]. These differences in the magnitude of influence of hydrocolloids on the rheological properties of gluten-free doughs seem to be related to the molecular structure and chain conformation of the polysaccharide that determine the physical intermolecular associations of the polymeric chain [39]. It could also be seen (Figure 1) that dough hardness increases with increase in the level of XG and LBG. The increase in the hardness of the dough could be due to the high water-binding capacity of gum long-chain polymers, which leads to scarcity of water for hydration [25].

### 3.4. Linear Viscoelasticity

**Figure 2:**
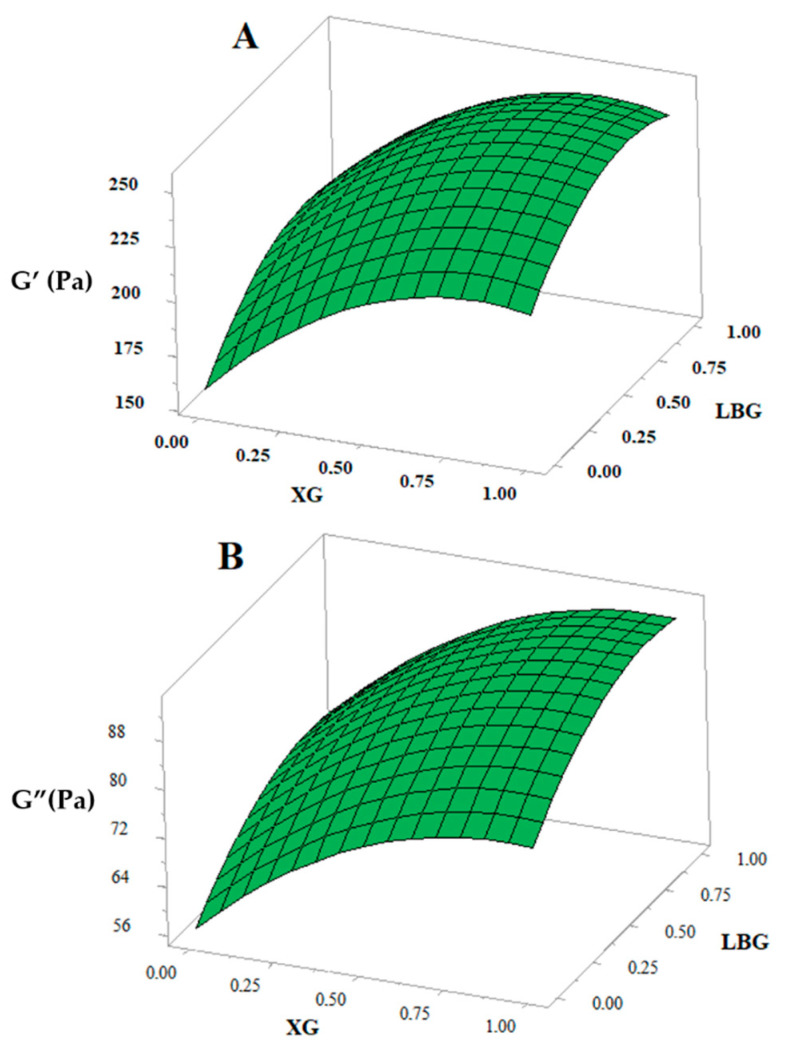
Response surface showing the effect of xanthan gum (XG) and locust bean gum (LBG) on linear viscoelastic properties (G’ and G”) of the dough.

The viscoelasticity of gluten-free dough formulations was examined by linear oscillatory measurements. Elastic or storage (G’) and viscous or loss (G”) modulus are significantly (*p* < 0.01, *p* < 0.001) affected by XG and LBG addition. The increase of gum concentration leads to increase of both modulus (G’ and G”) (Figure 2), with the effect of XG being the more pronounced (Table 4). These results are in accordance with the findings of Lazaridou et al. [27], who showed that addition of hydrocolloids to a rice-based dough resulted in a rise of elastic modulus (G’) as well as an increase in the resistance to deformation. They also reported that XG had a higher influence than the other gums used. The same trends have been reported by Turabi et al. [31] and Sabanis et al. [29] when several hydrocolloids were added to gluten-free formulations based on rice flour and corn starch—rice flour, respectively. They found that the highest apparent viscosity and consistency index values were obtained for doughs containing xanthan gum.

Compared to LBG supplementation, the highest elasticity of dough formulation supplemented by XG was associated with the weak gel properties and high viscosity values at low shear rates of aqueous xanthan gum dispersions due to its rigid and ordered chain conformation [39].

The values of G’ and G’’ at 1 Hz (Table 4) showed that for all gluten-free dough formulations, the elastic modulus (G’) was greater than the viscous modulus (G”) suggesting the predominance of the solid elastic-like behavior versus the viscous one.

At the interaction level, no significant effect of combined XG and LBG was exhibited on the rheological properties of doughs. Thus, an increase in the viscosity and strength of doughs could be due to the sum of their individual effects [23].

However, it was noticed throughout the gluten-free dough preparation process, that addition of gums brought a marked improvement in the mechanical handling properties of the dough for the different formulas tested. Lazaridou and Biliaderis [39] reported that supplementing of gluten-free formulations with hydrocolloids revealed an improvement in the viscoelastic properties of gluten-free doughs. Shittu et al. [12] have also reported that XG significantly increased the resistance of a composite dough to deformation.

### 3.5. Water Activity

Determining the water activity (a_w_) of food products is of great interest. It shows the availability of water for degradation reactions and thus helps predict their shelf life.

**Figure 3:**
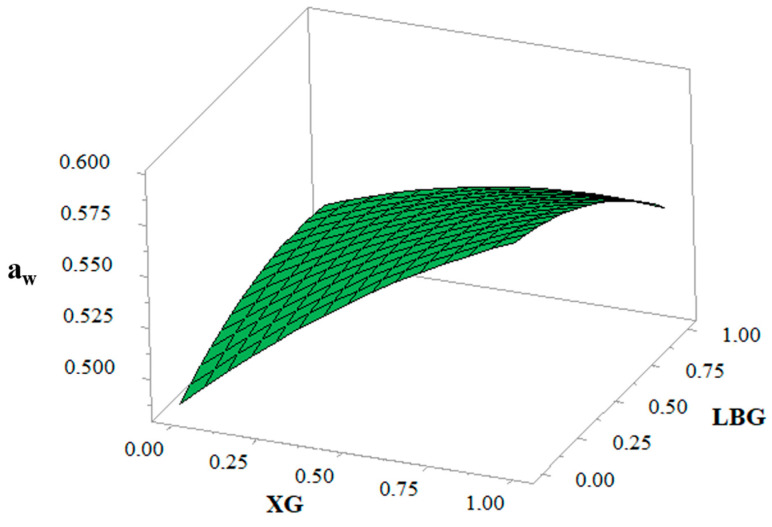
Response surface showing the effect of xanthan gum (XG) and locust bean gum (LBG) on the water activity (a_w_) of biscuits.

The results of regression analysis (Table 4) showed a positive effect of XG on the biscuit water activity, whereas a negative effect was exerted by LBG, with the effect of XG being more pronounced. Figure 3 confirmed that with the increase in the level of XG, a_w_ of the biscuit was significantly increased. The difference in the effect of each gum on the a_w_ of biscuits could be explained by their different affinities for water, which seems to correlate with the texture of the biscuits. It can be seen (Table 4) that higher a_w_ corresponds to softer biscuits.

Interaction between the gums showed a significant (*p* < 0.05) negative effect on the a_w_ of the biscuits. It could be due to the gel formation involving the association or cross-linking of the polymer chains to form a three-dimensional network that traps or immobilizes the water within it to form a rigid structure [19]. XG addition and its combination with guar gum in frozen bread dough reduced the freezable water amount and consequently the fusion enthalpy. Matuda et al. [40] showed that the combination of XG with guar gum in frozen bread dough resulted in higher reduction in the freezable water amount and consequently the fusion enthalpy during the frozen storage period.

### 3.6. Biscuit Specific Volome (Vsp)

Specific volume is one of the most important visual characteristics of cereal bakery products, strongly influencing consumers’ choice. Hence, it is a key parameter looked at when evaluating quality [20].

**Figure 4:**
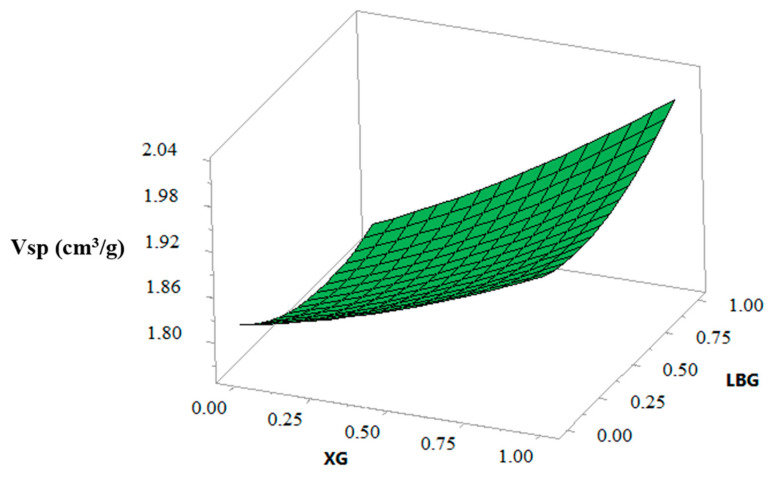
Response surface showing the effect of xanthan gum (XG) and locust bean gum (LBG) on the specific volume (Vsp) of biscuits.

The results of regression analysis depicted in Table 4 confirmed that a significant (*p* < 0.001) effect was found with both XG and LBG level, at linear and quadratic terms, on specific volume (Vsp) of biscuit with an increase in this parameter when the level of XG and LBG increase (Figure 4). Increase in Vsp of biscuits could be due to the high viscosity of the dough provided by the gums [30,31,41]. This viscosity arises predominantly from physical entanglement of conformationally disordered “random coils” when the concentration of the polymer is increased [19]. Consequently, the higher initial viscosity slows the rate of gas diffusion and favors the entrapment of air bubbles in the dough structure, thus allowing for improved retention at the early stage of baking [28,42]. It was also seen that the effect of Xanthan on Vsp of biscuits was more pronounced than that of LBG. Similar trends were obtained by Kaur et al. [26], Devisetti et al. [25], and Turabi et al. [31] when various hydrocolloids and gums were incorporated into buckwheat biscuits, proso millet cookies, and rice cakes, respectively. They indicated that the highest Vsp formulation was the one containing XG.

At the interaction level, a significant (*p* < 0.01) effect of combined XG and LBG on the Vsp of biscuits was observed (Table 4). The increase in the Vsp could be explained by their synergistic interaction involving thermoreversible gel formation, forming a viscoelastic three-dimensional network, which could be responsible for gas holding during baking [23]. According to Saha and Bhattacharya [19], solutions of XG or locust bean gum by themselves will not gel under any condition, but the combination will form firm gels. XG and polymannan chains associate following the xanthan coil-helix transition. Mixtures of XG and LBG require heating to about 95 °C to form a gel. For LBG the galactose deficient regions are involved in the association. They also reported that the interaction of XG with galactomannans is dependent on the ratio of the mixture, pH, and ionic environment, and the best synergism is obtained when gum ratios are 80/20 for guar gum/xanthan gum, 70/30 for konjac/xanthan, and 50/50 for LBG/xanthan gum.

### 3.7. Biscuit Hardness

**Figure 5:**
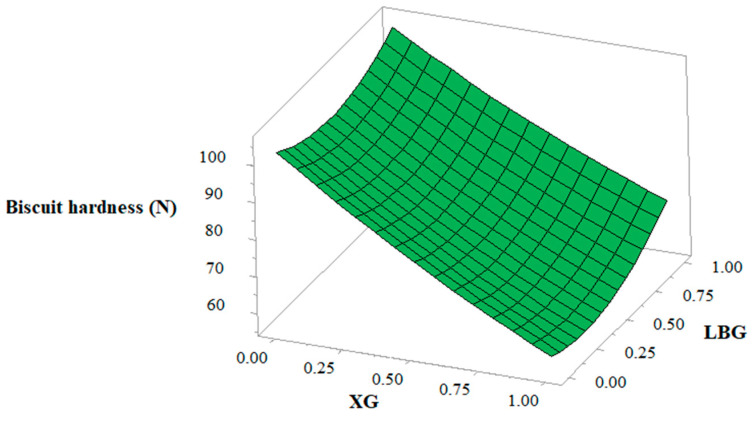
Response surface showing the effect of xanthan gum (XG) and locust bean gum (LBG) on biscuit hardness.

The coefficient of estimation of biscuit hardness (Table 4) showed that the level of XG and LBG had a significant effect especially at the linear level. XG had a negative effect on biscuit hardness, while LBG had a positive effect. In addition, biscuit hardness decreases as level of xanthan increases (Figure 5). These results are in accordance with the previous finding of Kaur et al. [26] where XG was incorporated into buckwheat biscuits. On the other hand, addition of LBG led to the contrary effect, and a significant increase (*p* < 0.05) in the hardness of the biscuits was observed as the level of LBG increased. Similarly to LBG, Sudha et al. [43] reported an increase in hardness with increase in the level of guar gum.

A negative correlation was observed between dough and biscuit hardness in the case of XG. On the contrary, LBG showed a positive correlation between both parameters. The softening effect of XG on the R–CPF biscuit texture might be associated with the greater moisture retention of the gum. A similar finding was reported by Benkadri et al. [24], when xanthan incorporation level was increased in the same biscuit formula. Kaur et al. [26] also showed similar trends for buckwheat biscuits incorporating various gums. They reported that addition of xanthan gum resulted in biscuits with maximum moisture retention and led to a decrease in the fracture strength of the biscuits. At the interaction level, no significant effect of combined XG and LBG was observed.

### 3.8. Optimization of Variables

The numerical optimization finds solutions corresponding to XG-LBG pairs that give the best compromise between the responses studied. It gives for each couple chosen the degree of desirability of each response studied, as well as the composite desirability.

An optimal formulation was chosen from the solutions suggested by the optimization software, having a XG and LBG level of 0.75% each. This formula has the higher desirability score (0.86) (Table 5).

A confirmative test for verification of the model was carried out using optimum levels of independent variables (0.75% for each XG and LBG gum). The confirmatory results (Table 5) show that the measured values of all the rheological parameters of the dough and physical parameters of the biscuit are close to the values predicted by the mathematical model without significant differences (*P* < 0.05) among them.

The results of the parameters measured for the optimal gluten-free formula based on R–CP were compared to those of the control gluten-free formula (R–CP) and wheat control (WF). The rheological parameters of the optimal gluten-free formula (hardness, G’ and G’’) were found to be significantly higher than those of the two control formulas.

Addition of the XG-LBG blend decreased the hardness of the biscuit from 110.77 N to 71.38 N, but the hardness remained greater than that of the control wheat biscuit (50.93 N). An improvement in the specific volume of 1.89 cm^3^/g was recorded against 1.68 cm^3^/g of the gluten-free control biscuit, but remained lower than that of the wheat control biscuit (2.35 cm^3^/g). However, an increase in the final water activity of the biscuits from 0.448 to 0.552 was noted after the addition of the gum blend, reflecting their water-holding capacity.

## 4. Conclusions

Response surface methodology was used to optimize the incorporation levels of xanthan (XG) and locust bean gum (LBG) for preparation of gluten-free biscuits based on rice–chickpea flour. All statistical terms (coefficient of determination R^2^, F-value and lack-of-fit test) revealed the statistical adequacy of the model. Regression analysis of the second order model revealed that linear terms of variables were more significant than quadratic terms on both dough and biscuit parameters, with the xanthan gum effect found to be more pronounced than LBG. Both xanthan and LBG exerted a positive effect on the rheological parameters of dough. XG showed a positive effect on the water activity of biscuits, which seems to correlate with the decrease in their hardness, exhibiting a softer texture. However, a negative effect on the water activity was exerted by LBG, leading to an increase in the hardness of the biscuits. Interaction terms showed a significant positive effect on the specific volume of the biscuits and a negative effect on the water activity, which could be explained by the synergistic effect between XG and LBG allowing the formation of a network of gels during cooking, mimicking the role of gluten in gas retention. However, the interactive effect of gums did not significantly affect the rheological parameters of the dough. Optimized conditions were developed to maximize the specific volume of biscuit and minimize water activity and biscuit hardness, while keeping hardness and viscoelastic properties of the dough in range. Optimum values for the formulation parameters, obtained via numerical optimization technique were founded 0.75% (based on composite flour weight) for xanthan and LBG. Therefore, predicted responses were found satisfactory for both rheological and physical characteristics of dough and biscuit.

## Figures and Tables

**Table 1:** Experimental design matrix for rice–chickpea composite flour (R–CPF) biscuit manufacture.

	Real Variables		Dough Parameters			Biscuit Parameters		
Expt N°	XG (%)	LBG (%)	Hardness (N)	G’ (Pa)	G” (Pa)	Hardness (N)	Vsp (cm^3^/g)	a_w_
1	0.15	0.15	21.50	195,300	69,970	91.31	1.81	0.520
2	0.85	0.15	32.84	234,825	84,580	62.73	1.91	0.580
3	0.15	0.85	29.96	214,967	76,977	93.62	1.80	0.530
4	0.85	0.85	38.76	251,550	92,210	69.29	1.95	0.550
5	0.00	0.50	24.56	190,300	66,773	98.95	1.76	0.500
6	1.00	0.50	41.56	245,875	90,973	57.65	1.94	0.574
7	0.50	0.00	26.57	204,156	71,978	78.89	1.87	0.543
8	0.50	1.00	34.74	240,050	88,400	86.44	1.87	0.530
9	0.50	0.50	31.73	243,050	85,215	74.64	1.84	0.545
10	0.50	0.50	31.84	241,650	85,750	74.66	1.84	0.556
11	0.50	0.50	32.75	240,967	86,540	75.49	1.83	0.544
12	0.50	0.50	29.90	253,050	88,695	78.26	1.85	0.554
13	0.50	0.50	32.77	231,500	85,570	75.29	1.84	0.553

XG: xanthan gum; LGB: locust bean gum; G’: elastic modulus; G’’: viscous modulus; Vsp: specific volume; a_w_: water activity).

**Table 2:** Ingredients of biscuits based on rice-chickpea composite flour (R-CPF) and wheat flour (WF) with varying xanthan gum (XG) and locust bean gum (LBG) gum levels, expressed in (%) (composite-flour weight basis).

Ingredients	R-CP Formula	WF Control Formula
Chickpea flour	78.13	/
Rice flour	21.87	/
Wheat flour (WF)	/	100.00
Hydrogenate vegetable fat	13.36	13.36
Sugar	18.12	18.12
Ammonium bicarbonate	0.93	0.93
Sodium bicarbonate	0.46	0.46
Salt	0.75	0.75
Xanthan gum (XG)	0.15–1.00	/
Locust bean gum (LBG)	0.15–1.00	/
Water	33.00 *	34.00 *

* Difference in the level of water addition in R-CPF and WF control formulation depended on the water absorption capacity of the flours of the two formulas (Benkadri et al., 2018).

**Table 3:** Analysis of variance (ANOVA) of the second order polynomial models for different properties of gluten-free doughs and biscuits containing xanthan gum (XG) and locust bean gum (LBG).

	Dough Parameters			Biscuit Parameters		
	Hardness (N)	G’ (Pa)	G’’ (Pa)	Hardness (N)	Vsp (cm^3^/g)	a_w_
Model (F-value)	43.94	24.64	48.54	144.11	179.75	22.09
Lack-of-fit (F-value)	1.29	0.34	2.66	1.08	0.79	2.38
*R^2^* %	96.91	94.62	97.20	99.04	99.23	94.04
*R^2^* % adj	94.71	90.78	95.19	98.35	98.68	89.78

**Table 4:** Regression coefficients of the second order polynomial models and significant terms for different properties of gluten-free doughs and biscuits containing xanthan gum (XG) and locust bean gum (LBG).

	Dough Parameters			Biscuit Parameters		
Terms	Hardness (N)	G’ (Pa)	G’’ (Pa)	Hardness (N)	Vsp (cm^3^/g)	a_w_
Intercept	31.799 ***	242,043 ***	86,354 ***	75.665 ***	1.841 ***	0.550 ***
XG	5.522 ***	19,338 ***	8008 ***	−13.915 ***	0.062 ***	0.023 ***
LBG	3.242 ***	10,894 **	4733 ***	2.442 **	0.003	−0.005 *
XG^2^	0.358	−10,962 **	−3390 **	1.008	0.005	−0.005
LBG^2^	−0.845	−8954 **	−2732 **	3.190 **	0.017 ***	−0.005 *
XG LBG	−0.637	−735	156	1.060	0.014 **	−0.010 *

* Significant at *p* < 0.05, ** significant at *p* < 0.01, *** significant at *p* < 0.001.

**Table 5:** Predicted and measured values of the rheological and physical parameters of the optimum selected formula compared to control (R-CPF and WF) formulas (% flour weight-based).

	Variables		Dough Parameters			Biscuit Parameters			
	XG (%)	LBG (%)	Hardness (N)	G’ (Pa)	G” (Pa)	Hardness (N)	Vsp (cm^3^/g)	a_w_	D
Predicted values	0.75	0.75	37.43	253,095	92,380	70.18	1.90	0.553	0.86
Measured values	0.75	0.75	39.44 (±0.59)	253,450 (±1768)	90,430 (±3168)	71.38 (±8.95)	1.89 (±0.05)	0.552 (±0.002)	
R–CP Formula			20.43 (±1.16)	139,800 (±4950)	48,550 (±3069)	110.77 (±6.86)	1.68 (±0.008)	0.448 (±0.003)	
WF Formula			13.61 (±0.53)	95,455 (±1450)	32,670 (±2079)	50.93 (±2.70)	2.35 (±0.006)	0.550 (±0.004)	

Vsp: specific volume, D: desirability, R-CP: rice–chickpea, WF: wheat flour.

## Data Availability

Data Availability Statements in section “MDPI Research Data Policies” at https://www.mdpi.com/ethics.
